# Influence of nutrient enrichment on temporal and spatial dynamics of dissolved oxygen within northern temperate estuaries

**DOI:** 10.1007/s10661-021-09589-8

**Published:** 2021-11-15

**Authors:** MRS Coffin, KM Knysh, SD Roloson, CC Pater, E Theriaul, JM Cormier, SC Courtenay, MR van den Heuvel

**Affiliations:** 1 Fisheries and Oceans Canada, Gulf Fisheries Centre, 343 University Ave, Moncton, NB E1C 5K4 Canada; 2 Department of Biology, Canadian Rivers Institute, University of Prince Edward Island, Charlottetown, Canada; 3 Canadian Water Network, School of Environment, Resources and Sustainability, Canadian Rivers Institute, University of Waterloo, Waterloo, Canada

**Keywords:** Nutrient, Oxygen, Spatial variation, Tide, Temperature, Eutrophication

## Abstract

In temperate estuaries of the southern Gulf of St. Lawrence, intermittent seasonal anoxia coupled with phytoplankton blooms is a regular occurrence in watersheds dominated by agricultural land use. To examine the spatial relationship between dissolved oxygen and phytoplankton throughout the estuary to assist in designing monitoring programs, oxygen depth profiles and chlorophyll measurements were taken bi-weekly from May to December in 18 estuaries. In five of those estuaries, dissolved oxygen data loggers were set to measure oxygen at hourly intervals and at multiple locations within the estuary the subsequent year. The primary hypothesis was that dissolved oxygen in the upper estuary (first 10% of estuary area) is predictive of dissolved oxygen mid-estuary (50% of estuary area). The second hypothesis was that hypoxia/superoxia in the estuary is influenced by temperature and tidal flushing. Oxygen depth profiles conducted in the first year of study provided preliminary support that dissolved oxygen in the upper estuary was related to dissolved oxygen throughout the estuary. However, dissolved oxygen from loggers deployed at 10% and 50% of estuary area did not show as strong a correlation as expected (less than half the variance explained). The strength of the correlation declined towards the end of summer. Spatial decoupling of oxygen within the estuary suggested influence of local conditions. Chlorophyll concentration seemed also to be dependent on local conditions as it appeared to be coupled with the presence of sustained anoxia in the upper estuary with blooms typically occurring within 7 to 14 days of anoxia. The practical implication for oxygen monitoring is that one location within the most severely impacted part of the estuary is not sufficient to fully evaluate the severity of eutrophication effects.

**Supplementary information:**

The online version contains supplementary material available at 10.1007/s10661-021-09589-8.

## Introduction

The impacts of nutrient enrichment on estuaries are well-characterized: increased biomass of primary producers and displacement of seagrass by macroalgae and phytoplankton followed by the development of hypoxia/anoxia (D’Avanzo et al., 1996; Diaz & Rosenberg, 2008; Valiela et al., 1997). The most common indicators for quantifying eutrophication in estuaries are chlorophyll *a*, a proxy for phytoplankton (Meeuwig, 1999), seagrass health metrics (Hitchcock et al., 2017; Short et al., 2006), or seagrass coverage (van den Heuvel et al., 2019). However, in shallow systems that are impacted by nutrients, the relative proportion of primary production can be skewed towards benthic macroalgae rather than pelagic production (Lavaud et al., 2020; Valiela et al., 1997) meaning that no single primary production-based indicator is sufficient. Furthermore, all primary producers eventually decompose, releasing nutrients for further growth and increasing oxygen demand. Given that dissolved oxygen is produced via photosynthesis and consumed through respiration, it has been suggested that dissolved oxygen represents an estimate of overall ecosystem metabolism (Caffrey et al., 2004). For these reasons, dissolved oxygen has been used as a monitoring endpoint in shallow, nutrient-impacted estuaries (Coffin et al., 2018a, b; D’Avanzo et al., 1996; Iriarte et al., 2010).

Dissolved oxygen concentration in estuaries is influenced by a variety of processes including photosynthesis, respiration, and transport through tidal processes (Iriarte et al., 2015; Lake & Brush, 2015; Murrell et al., 2018). Hypoxia develops when the consumption of oxygen exceeds production and/or import and can vary dramatically throughout days and seasons because of these competing processes (Lake & Brush, 2015). Land-derived nutrients enter estuaries via freshwater and promote increased primary productivity. Ocean-water is usually nutrient-poor, and oxygen saturated, ocean and freshwater mix throughout the estuary resulting in heterogenous concentrations of both (Iriarte et al., 2015; Valiela et al., 2021). Few studies have examined spatial or temporal/seasonal dissolved oxygen patterns in shallow, small- to medium-sized estuaries, due to the complex nature of the systems and high variability within and among estuaries (Iriarte et al., 2015).

 The accepted paradigm for explaining nutrient uptake spatially is that primary production from anthropogenic nutrient enrichment is generally greatest at the upstream portion of the estuary where nutrients are first available to estuarine and coastal systems (Paerl et al., 2014; Valiela et al., 2021). However, there is a poor understanding of how dissolved oxygen concentration varies along the longitudinal axis, e.g., nutrient and salinity gradients that are present in estuaries. For example, a study in Spain demonstrated that temperature, primary productivity, river discharge, and tides were all significant factors affecting dissolved oxygen but that the strength of those effects varied among estuaries and further varied within an estuary (Iriarte et al., 2010, 2015).

The seasonal or temporal nature of dissolved oxygen and/or nutrient-related hypoxia can vary dramatically with climate, location, and geographic features (Zhang et al., 2010). Temperate estuaries have distinct seasons and periods of nutrient supply which manifests as more seasonally variable nutrient uptake and primary production compared to estuaries at lower latitudes, although pathways of effect are similar (Teichberg et al., 2010). In northern, icebound, temperate estuaries, the period of eutrophic conditions resulting in hypoxia can be limited to only days or weeks (Coffin et al., 2018a, b) compared to estuaries with longer periods of susceptibility for hypoxia (Zhang et al., 2010).

The purpose of this study was to examine the spatial and temporal dissolved oxygen dynamics within and among temperate, typically microtidal, winter ice-bound estuaries of the southern Gulf of St. Lawrence to understand the minimum required monitoring design to capture spatial variability. A monitoring program of this nature is critical for management decision-making pertaining to ecosystem health and shellfish aquaculture. The hypothesis was that nutrient impacts, assessed via dissolved oxygen and chlorophyll patterns, are most severe in the upper estuary and that, while impacts dampen with distance downstream, they are felt throughout the estuary such that data collected from a single location will be positively correlated to data collected throughout an estuary. Secondly, it was hypothesized that both temperature and tidal amplitude influence the manifestation of hypoxia more strongly in eutrophic than mesotrophic estuaries. This is important from a monitoring context in understanding how different estuaries respond differentially to nutrient loads for multi-estuary models (e.g., DFO, 2021). Hypotheses were tested by surveying water column dissolved oxygen from the upper to the outer estuary and with the collection of high-frequency continuous oxygen measurements collected in the upper and midpoint of an estuary. In addition, tidal models based on direct observations were employed to assess temporal differences in flushing rate on dissolved oxygen concentrations.

## Materials and methods

### Study area

Estuaries in the southern Gulf of St. Lawrence are generally small, shallow, with similar watershed sizes and shared lithology throughout much of its basin (Grizard et al., 2020; Slaymaker et al., 2020). Low freshwater input from these relatively small watersheds (17–386 km^2^ in this study) leads to well-mixed estuaries throughout the water column and a short transition zone from fresh to salt water—particularly the area from 0 to 15 PSU (DFO, 2021). Historically, eelgrass (*Zostera marina* L.) has dominated estuaries within the southern Gulf of St. Lawrence but is undergoing decline for decades (Hanson, 2005; van den Heuvel, 2019), largely linked to effects of eutrophication, particularly in estuaries of PEI (Bugden et al., 2014; DFO, 2009) where land use is intensely agricultural (Grizard et al., 2020; Jiang et al., 2015). This increased nutrient availability, primarily from nitrate-based fertilizers (Grizard et al., 2020), has led to inundation by the green macroalga *Ulva* spp. (primarily *U. lactuca* (L.)) in the upper estuary defined here as the area of salinity 15–20 PSU that typically occurs in the first 10–25% of estuary area. Southern Gulf of St. Lawrence estuaries are typically ice-covered from January to mid-April, although the period of ice coverage has been in decline in recent years (Manson et al., 2016).

**Fig. 1:**
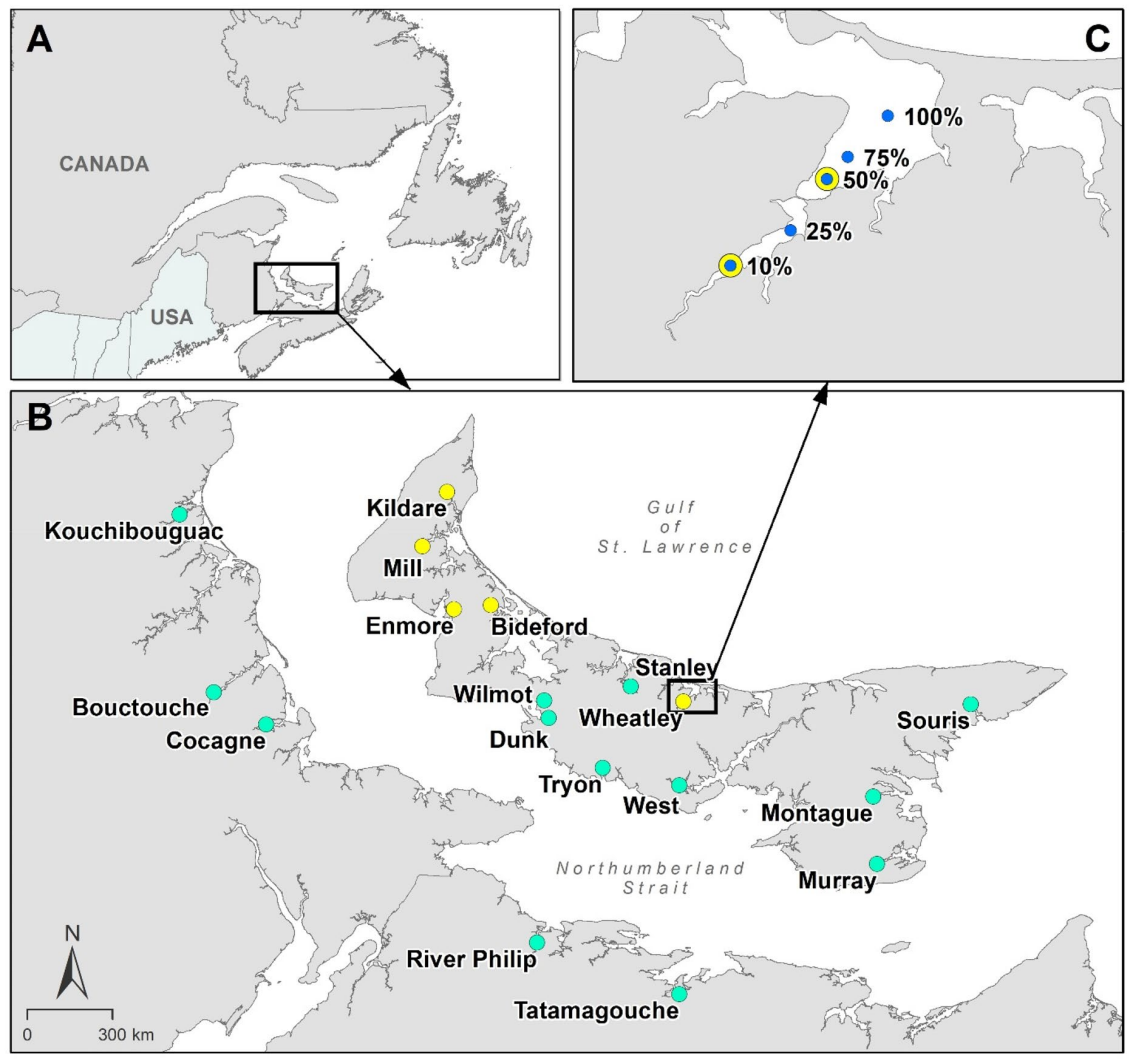
A Study area in the context of northeastern North America. B Study sites located in the southern Gulf of St. Lawrence. Green circles indicate study sites where depth profiles for water chemistry were taken in 2013. Yellow circles were also monitored in 2013 but in 2014 had dissolved oxygen loggers. C The blue circles indicate the approximate location for oxygen depth profiles conducted in 2013 in an example estuary

Eighteen estuaries, spanning a broad nutrient loading gradient and also the geographic extent of the southern Gulf of St. Lawrence (Canada), were selected for study in 2013 (May to October, Fig. 1; Table 1; see Coffin et al. (2018a, b) for more information about these sites). Tidal amplitudes in this region are diurnal or semi-diurnal, sometimes both within an estuary, depending on time of year (Davidson-Arnott, 2006; Pingree & Griffithis, 1980) and averaged between 0.3 and 1.6 m, among estuaries in this study. Five PEI estuaries, a subset of the 18 estuaries studied in 2013, were studied in 2014. The five estuaries were chosen based on low nutrient impact (Bideford and Enmore) or high nutrient impact (Kildare, Mill River and Wheatley), according to data from 2013 and Coffin et al. (2018a, b). Specifically relative impact was based on the relative frequency of hypoxia and oxygen superoxia as well as the dominant vegetation, *Z. marina* in low nutrient impact estuaries, and *Ulva* spp. in high nutrient impact estuaries. Within the southern Gulf of St. Lawrence, there are no estuaries, to our knowledge, that are eutrophic and are dominated by phytoplankton instead of *Ulva* spp., i.e., as described in Valiela (1997). Dissolved oxygen loggers were deployed at the midpoint of transects delineating the upper 10% of estuary area and 50% of estuary area according to Coffin et al. (2018a, b) as determined from estuary polygons using ArcGIS software (Redlands, CA, USA; Fig. 1).

**Table 1:** Watershed characteristics for the eighteen watersheds sampled in 2013 (*Z Zostera marina*, *U Ulva* spp., *B* Bare; adapted from Coffin et al. (2018b)), presented by declining Nitrate–N loading

Site	Watershed area (km^2^)	Water residence (d)	Nitrate–N (kg/ha/yr)	Dominant vegetation
Wilmot	71.6	0.73	427.2	U
Dunk	161.1	0.76	312.5	U
Tryon	41.85	0.24	214.7	B
Montague	163.8	2.17	175.6	U
Wheatley	42.1	1.74	149.0	U
Mill	88.3	2.72	118.8	U
Tatamagouche	225.6	0.35	52.4	Z
Kildare	17.4	3.49	49.6	U
Souris	31.6	1.85	49.1	U
Stanley	39.2	3.80	42.5	U
Murray	57.25	2.40	40.9	U
West	113.6	0.82	29.9	B
Kouchibouguac	385.7	1.37	6.4	Z
Enmore	36.6	0.56	5.8	Z
Bouctouche	376.7	1.16	1.7	Z
Bideford	19.3	2.13	1.4	Z
Cocagne	248.8	1.19	0.8	Z
River Philip	70	No data	1.20	Z

### Water chemistry, chlorophyll, bathymetry, and water residence

Depth profiles of water chemistry variables were taken bi-weekly in 2013, May 27 to October 30, using a YSI V2 6600 multi-parameter sonde (Yellow Springs, OH, USA) with an optical probe for dissolved oxygen and additional probes for pH, conductivity, depth, and temperature. Measurements were taken every 0.5 m within the water column of the primary channel, from 0.5 m above the substrate to 0.5 m below the water’s surface, at five locations within each of the 18 estuaries studied in 2013. The upper boundary of the estuary was determined by mean salinity greater than 0.5 PSU. The outermost extent was determined by geography and salinity, where the estuary opens into a coastal embayment or the Northumberland Strait directly and fresh and salt water were completely mixed. Using these criteria, five sites were sampled along each estuary positioned near the midpoint of transects corresponding to 10, 25, 50, 75, and 100% of the estuary area (Fig. 1). To theoretically capture dissolved oxygen at its lowest point during the day, water quality measurements were taken between dawn and 11 AM and always within 2 days of the neap tide (when tidal flushing is lowest). Bathymetric data and water residence time were collected and calculated using the same methodology presented in Coffin et al. (2018a, b) and are the same except for the addition of two additional sites (Murray River, PE and Cocagne, NB).

Chlorophyll samples were collected in 1 L brown bottles at the 10% and 50% stations at a depth of 0.5 m below the water’s surface. Samples were stored on ice for less than 6 h prior to filtering upon return to the laboratory through GF/F Whatman glassfibre filter papers (0.7 µm). Filter papers were stored in 5 mL of 100% acetone at − 80 °C until analysis. Extracted chlorophyll contained in the acetone was filtered again through a 0.45-µm syringe filter and immediately analyzed for chlorophyll *a* and chlorophyll *b* using high-performance liquid chromatography. Total chlorophyll is presented. Analyses were conducted using a Varian model 240 pump, model 410 autosampler, model 335 diode array detection, and model 363 fluorescence detector. Samples were run through an Agilent Technologies Zorbax Eclipse Plus C18 reverse phase column (250 × 4.6 mm, 5-μm particle size), with a column oven temperature of 30 °C. The mobile phase consisted of 5% 0.5 M ammonium acetate, 15% methanol, and 80% acetonitrile changing to 20% methanol and 80% acetonitrile over 5 min and held for an additional 10 min at a flow rate of 1 mL/min. Chlorophyll was quantified against pure standards of chlorophyll *a* and *b* (Sigma, St. Louis, USA), either at a fixed absorbance wavelength of 430 nm or using 430/650 fluorescence excitation and emission wavelengths. Absorbance was only used for quantification in the rare (< 2%) instances where the fluorescence reading was beyond the linear range. Chlorophyll was expressed as total chlorophyll concentration (µg/L), the sum of all chlorophyll pigments measured.

### Continuous oxygen monitoring

In 2014, Onset Hobo® Dissolved Oxygen loggers (Bourne, MA, USA), using optical sensor technology and equipped with copper anti-fouling caps, were set to record dissolved oxygen (mg/L) and temperature (°C) every hour in the estuaries of the Bideford, Enmore, Kildare, Mill, and Wheatley Rivers (Fig. 1). Loggers were deployed 0.5 m from the substrate, in each upper estuary location and at both 0.5 m below the water’s surface and 0.5 m above the substrate at the 50% location. Loggers deployed near the substrate were moored to a steel pole embedded in concrete, whereas loggers deployed immediately below the water’s surface were attached to a rope 0.5 m below a tethered buoy. Data from dissolved oxygen loggers were downloaded, and loggers were cleared of fouling weekly, May to December 2014.

### Statistical analysis

Interpolated dissolved oxygen data, incorporating oxygen against depth and time, are presented as rasters for every study site with each pixel in the raster representing an oxygen datum point. Interpolations were produced using SigmaPlot V 11.2 and then analyzed using ArcGIS v10.5. Hypoxia, dissolved oxygen concentration < 2 mg/L and represented by red pixels, was characterized as the relative proportion of red pixels to other colors for all locations within an estuary using pixel-based image analysis. Each individual pixel within a raster represents 1.41 days on the x-axis and 1.1% of the total depth on the y-axis; the y-axis is depth standardized due to variance in water depth related to tides at the time of data collection (substrate to surface = 0 to 100%). Total hypoxia present within the depth profiles is then presented in descending order of severity (left to right).

**Fig. 2:**
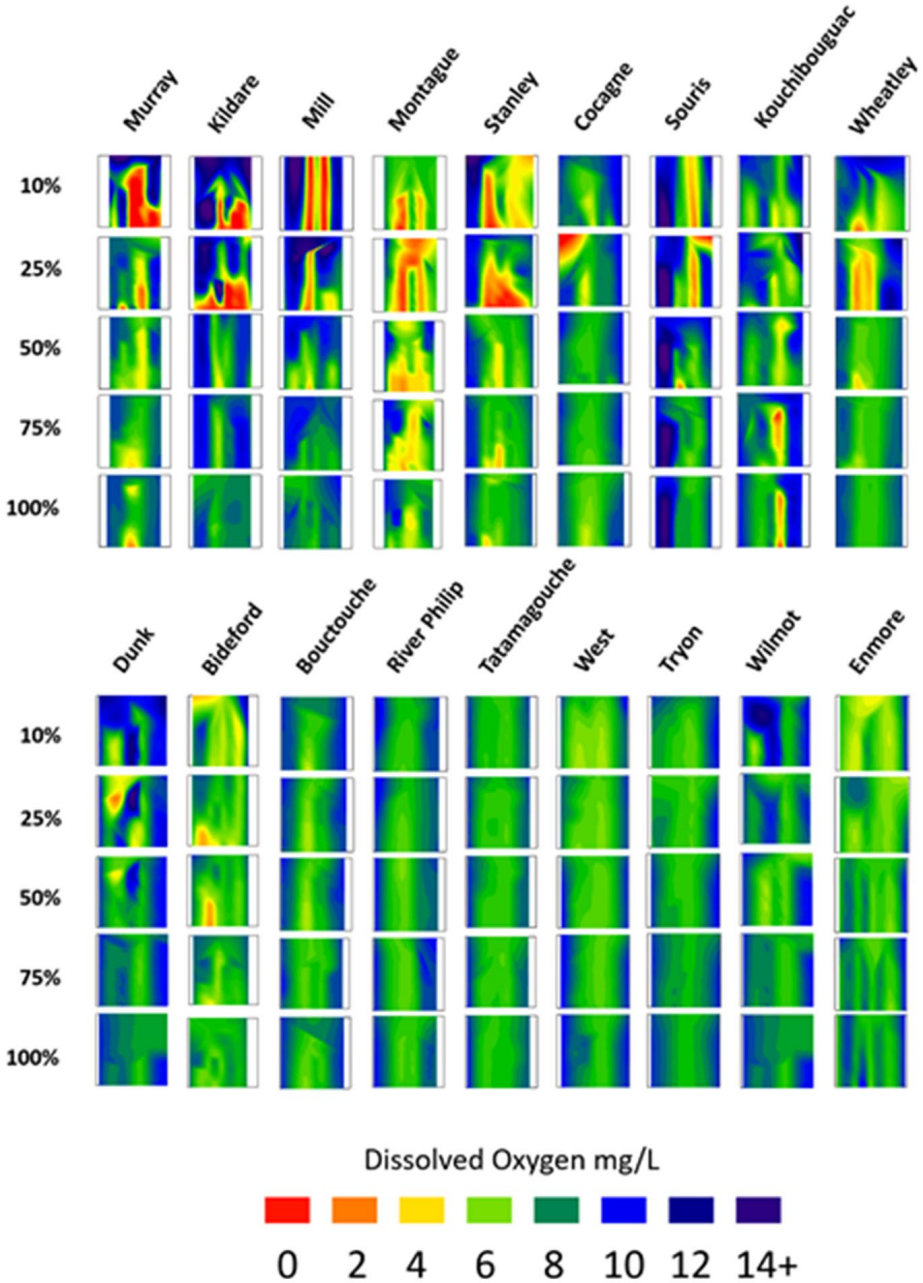
Dissolved oxygen from all estuaries in 2013 represented as a contour plot with depth on the y-axis, time on the x-axis (May 27 to October 30, 2013), and oxygen indicated by color. Rows are estuary area from the upper estuary (10% being the upper estuary and 100% being the boundary with the ocean) where dissolved oxygen was routinely measured. Columns are sites from Fig. 3.1. They are ordered to emphasize the gradient of impact along the estuary and, from left to right, the most severe anoxia first

To compare the continuous dissolved oxygen data among estuaries and within different parts of the estuary, oxygen data were standardized to account for dissolved oxygen solubility which is inversely related to salinity, pressure, and temperature. The average salinity (Fig. 2) was used to correct for salinity, and a standardized pressure was assigned for each logger dataset. Since temperature fluctuates dramatically throughout the year (~ 0–30 °C), all data were corrected to 15 °C for dissolved oxygen solubility in water according to the equations found in Weiss (1970) and Garcia and Gordon (1992), which were adapted to a python script (Python v2.7). Tidal amplitude was determined using harmonic analysis using the t tides MATLAB script (Pawlowicz et al., 2002) based on measurements taken every 10 min for 30 + days using Onset Hobo Water Level Titanium® loggers (see Coffin et al. (2018a, b) for more detailed methodology). All oxygen, temperature, and tidal amplitude ranges were calculated on a daily basis as this best represented the spring to neap tide transitions. To account for time series-related effects that may not have been explained by temperature and tide, Julian day was used as a dependent variable. Chlorophyll was compared to metrics of oxygen < 4 mg/L calculated for 14 days, 7 days, and week starting prior to each chlorophyll measure. The threshold of 4 mg/L was selected rather than the 2 mg/L threshold due to insufficient data below 2 mg/L for some estuaries that precluded comparison. Correlations of oxygen and chlorophyll data were conducted in Statistica V.13 Software.

Generalized Additive Mixed-Modeling (GAMM) was used to examine the daily oxygen relationship with tidal height, temperature, and interactive predictors using the R statistical programming platform and the package *mgcv* (Iriarte et al., 2010; R Core Team, 2020; Wood, 2017). As spatial autocorrelation, and water connectivity, between upper and outer estuary logger varied with time, individual loggers were treated individually for time-series predictions similar to the estuarine oxygen analysis in Iriarte et al. (2010). Smoothing relationships within the GAMM’s employed penalized cubic regression splines assuming a Gaussian response (Iriarte et al., 2010; Wood, 2017). Julian day smoothers used cyclic-cubic basis function to account for seasonality, whereas other environmental predictors used a cubic basis (Wood, 2017). Final maximum-likelihood model selection started with a full interaction structure of Julian day, temperature, tidal height, and two-way and three-way interaction terms with backwards selection dropping the highest approximate *p*-value until only smoothed terms with *p* < 0.05 remained in the model (Wood, 2017). Residual normality and heterogeneity were visually assessed using quantile–quantile (QQ) plots and fitting deviance residuals against linear predictors. Gaussian assumption violations of a hypoxia-inflated and bimodal site are noted. A first-order autoregressive model (AR(1)) structure was applied to the model to account for temporal autocorrelation in oxygen responses (Iriarte et al., 2010; Wood, 2017). Autocorrelation function (ACF) plots of models fitted with and without AR(1) checked if temporal autocorrelation was reduced.

## Results

### Spatial patterns of dissolved oxygen

In estuaries that experienced hypoxia/anoxia, dissolved oxygen concentration increased between the 10 to 100% area locations (first ten estuaries in Fig. 2, except Kouchibouguac River in Kouchibouguac National Park, which had the lowest dissolved oxygen in the outer estuary). Estuaries that did not experience hypoxia (last eight estuaries in Fig. 2) showed the same pattern, but the magnitude of the difference along the axis of the estuary was much less. In estuaries experiencing low oxygen, anoxia was mostly restricted to the 10% and 25% locations (upper estuary) where it occurred earlier in the season and for longer durations than sites farther out. Dissolved oxygen concentration was typically lower near the substrate and higher closer to the water’s surface (Fig. 2). In total, upper estuary anoxia was detected at 10 sites, eight of which were located on Prince Edward Island (Fig. 2). Of the two sites in New Brunswick that experienced anoxia, Kouchibouguac and Cocagne, only one was in the upper estuary (25% location for Cocagne and near the water’s surface versus 75% and 100% locations for Kouchibouguac which is a coastal lagoon), but these anoxic events did not persist beyond a single punctual sampling point.

### Temporal pattern of dissolved oxygen

**Fig. 3:**
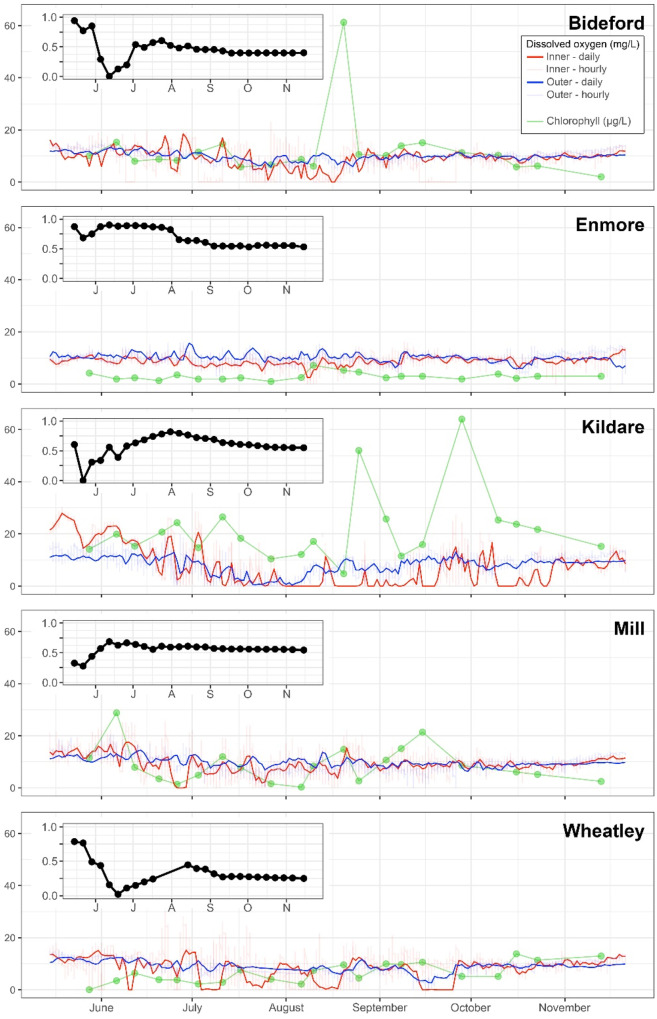
Dissolved oxygen concentration (mg/L) from both the 10 and 50% location in each study estuary where loggers were deployed (Bideford, Enmore, Kildare, Mill, and Wheatley) is presented. Dissolved oxygen concentration is presented raw using semi-transparent red and blue lines for the 10 and 50% locations, respectively, and as a daily average (over the daily tidal cycle), as darker red and blue lines. Weekly correlations of the averaged daily tidal cycle dissolved oxygen were then correlated, 10% dissolved oxygen with 50%, and presented within the inset in the top left corner. Finally, green circles connected with straight green lines represent point samples for total chlorophyll measured in µg/L at the 10% location

At nutrient-impacted sites, dissolved oxygen was quite variable within short periods, ranging from superoxic (30 mg/L) to anoxic within 24 h at the 10% location (Fig. 3). Similar to the depth profiles in 2013, dissolved oxygen logger data in 2014 showed that hypoxia was more prevalent at 10% relative to 50% (Fig. 3). In estuaries where anoxia occurred, anoxia happened earlier in the year at the 10% location and lasted longer into the fall when compared to the 50% location (Fig. 3), generally corroborating what was observed with bi-weekly depth profiles the previous year (Fig. 2). Furthermore, anoxia was rarely observed at the 50% location at all sites except for Kildare which was impacted at both the 10 and 50% locations (Fig. 3, Table 2). Dissolved oxygen was considered related to eutrophication when it fell outside of oxygen concentrations in the 4–10 mg/L range, and this metric was termed “eutrophic time” (Coffin et al., 2018a, b; Table 2). In Mill River, the loggers (top and bottom) at the 50% location were lost, due to a boat strike, resulting in the loss of data from mid-July to early-August after which only a “bottom” logger was deployed. There was no top logger at Enmore as the water was too shallow to discriminate between top and bottom at low tide (~ 0.5–1.0 m).

**Table 2:** Dissolved oxygen metrics for each of the loggers deployed in 2014 over the entire deployment period. The first three metrics are the proportion of the entire deployment that dissolved oxygen concentration (mg/L) was above or below the indicated thresholds. Spearman correlation coefficients between upper and outer logger locations and the two outer loggers (top/bottom) are also presented for each site. Loggers at 50% top were lost at Enmore and Mill. Sites are again ordered by declining Nitrate–N loading, presented here again for reference

Site	Location	< 4	> 10	< 4 + > 10	Mean	Coefficient of variation	Correlation 10% vs. 50% bottom	Correlation 50% top vs bottom	Nitrate–N (kg/ha/yr)
**Wheatley**	10%	0.1	0.41	0.51	9.32	0.46	0.64	0.70	149.0
	50% top	0	0.45	0.45	9.79	0.23			
	50% bottom	0.02	0.38	0.4	9.29	0.31			
**Mill**	10%	0.13	0.47	0.6	9.29	0.47	0.25	N/A	118.8
	50% bottom	0.03	0.38	0.41	9.24	0.29			
**Kildare**	10%	0.46	0.34	0.8	7.54	1.08	0.55	0.66	49.6
	50% top	0.02	0.7	0.72	11.33	0.26			
	50% bottom	0.16	0.42	0.58	8.33	0.48			
**Enmore**	10%	0.02	0.28	0.3	8.82	0.25	0.53	N/A	5.8
	50% bottom	0	0.5	0.5	9.9	0.2			
**Bideford**	10%	0.21	0.42	0.63	8.24	0.57	0.4	0.2	1.4
	50% top	0	0.23	0.23	9.09	0.15			
	50% bottom	0	0.49	0.49	9.92	0.23			

While depth profile data collected in 2013 suggested a strong spatial correlation for dissolved oxygen throughout the estuary (based on the interpolations contained in Fig. 2), in 2014 oxygen at 50% of estuary area, measured 0.5 m from the substrate, only correlated with values from the 10% oxygen logger about half of the time (Fig. 3). Of the three sites with the full complement of dissolved oxygen loggers (Bideford, Kildare, and Wheatley), the 50% bottom logger was better correlated with the 10% logger than the 50% top logger with the exception of Bideford. Wheatley had the strongest correlation between the two 50% loggers (Table 2). There was no pattern in the strength of correlation between 10 and 50% locations based on the level of nutrient impact. At all sites, the correlation between the 10 and 50% bottom locations changed throughout the field season. All five estuaries were well correlated initially with the correlation coefficient between 10 and 50% bottom loggers decreasing in May to June and then generally increasing into summer before stabilizing at the highest point around the beginning of August (Fig. 3). The correlation coefficient decreased after this indicating decoupling between the 10 and 50% locations until the end of the field season (Fig. 3).

### Chlorophyll oxygen relationships

As with oxygen, chlorophyll from the 10% location showed that Kildare was the most eutrophic with peaks in total chlorophyll above 50 µg/L (Fig. 3). This was followed by Wheatley, and Mill River, with Bideford and Enmore showing the lowest chlorophyll levels. Chlorophyll in the inner estuary tended to be higher than the outer estuary (slope of the 50% vs. 10% regression varied between 0.05 and 0.22 for all estuaries). The differences between 10 and 50% chlorophyll were only statistically different at Bideford and Kildare as determined with paired t-tests. There was no consistency in timing of chlorophyll peaks between estuaries, but the highest concentrations occurred between May and October, depending on the estuary. Statistically significant correlations between chlorophyll concentration and anoxic time were found when anoxic time was calculated for the previous 7 days, 14 days, and for the previous 7 days starting a week prior to the chlorophyll measurement. The strongest overall correlation with chlorophyll was with the anoxic time for the week ending 7 days prior to the chlorophyll measurement (*r* = 0.39). On an individual estuary basis, those correlations were only statistically significant for the Mill and Enmore estuaries.

### Influence of temperature and tidal amplitude on dissolved oxygen

GAMM models conducted individually at 10% and 50% bottom stations showed complex patterns of effect and interaction of Julian day, temperature, and tide. The patterns of effect differed both with degree of nutrient impact and the position in the estuary. For the 10% stations, the more impacted estuaries Wheatley, Mill, and Kildare showed additive Julian day and temperature effects or a Julian day-temperature interaction (Table 3). Conversely, the Enmore 10% location showed temperature and tide main effects, while Bideford 10% had a tide-temperature-Julian day interaction (Table 3). The key difference is the presence or absence of the tidal effects. The less severely nutrient-impacted 50% stations also showed a complex pattern of effects, though Julian day was the only effect at Bideford, Wheatley, and Kildare, while temperature was the only effect at Enmore (Table 3).

**Table 3:** General Additive Mixed Model (GAMM) results with oxygen (corrected to 15 °C) as the dependent variable and Julian day, temperature, and tidal amplitude range as the independent variables. Numbers refer to effective degrees of freedom (EDF) of model terms to indicate the degree of non-linearity, with a value of 1 indicating a linear relationship and values > 1 indicating more complex relationships between parameters. Only significant or best model terms are listed here, all other GAMM results are found within S1

Site	Julian day		Temperature		Tidal amplitude		Interaction	
	10%	50%	10%	50%	10%	50%	10%	50%
Wheatley	4.6	5.5	1.9					
Mill	6.4	1.9	4.3			2.6		
Kildare^1^		5.3					Julian day*Temperature -7.8	
Enmore			1	1	1			
Bideford^2^							Julian day*Temperature*Tide-3.7	

^1^Best fitting Gaussian model of hypoxia-inflated data. See Fig. 4b

^2^*p* = 0.069, best fitting Gaussian model of bimodal data. See Fig. 4b

**Fig. 4:**
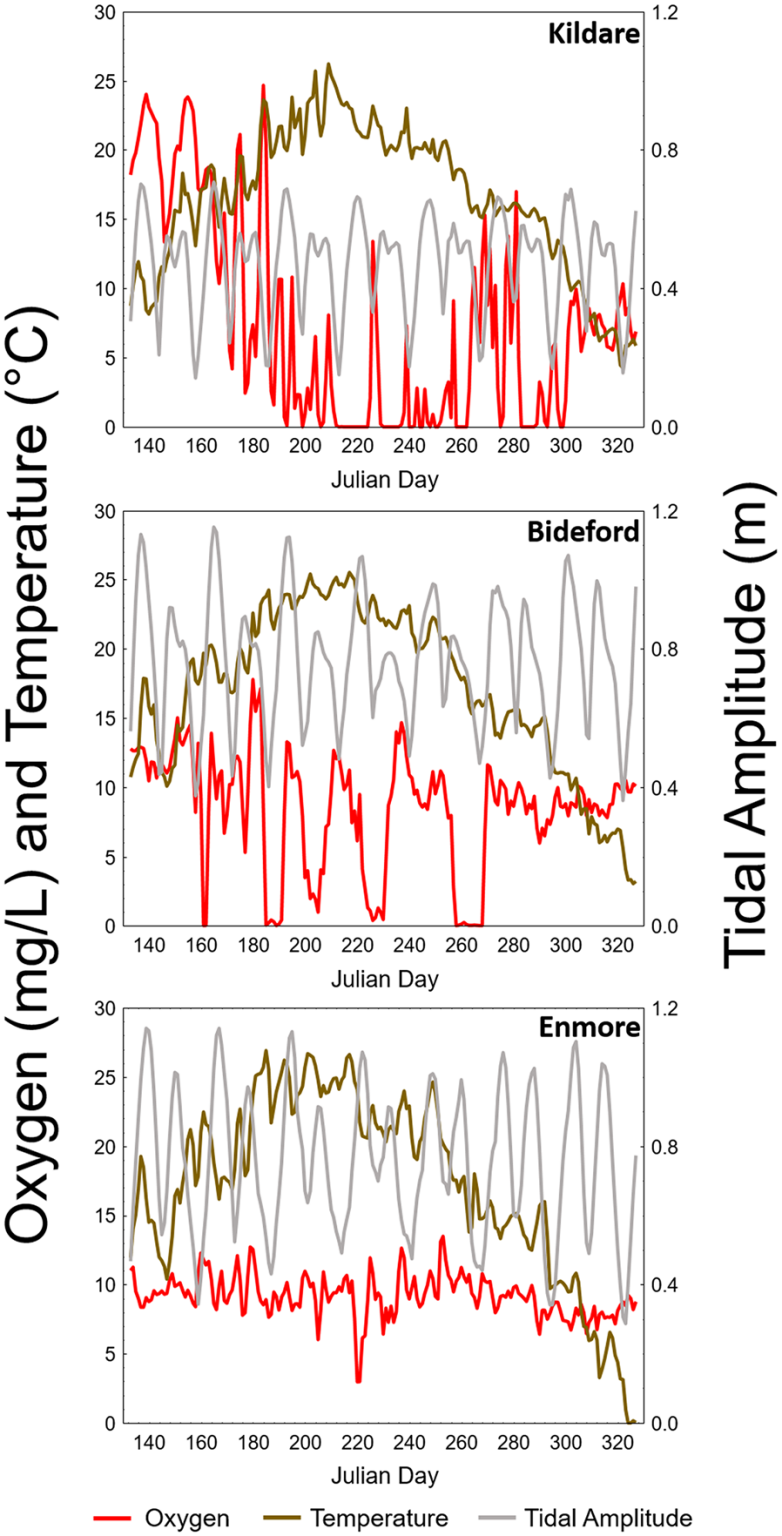
Raw daily mean oxygen (mg/L corrected to 15 °C), mean temperature (°C), and tidal amplitude range (m) for Kildare 10% location, Bideford 10% location, and Enmore 10% location, top-to-bottom

Graphical representations of the raw oxygen, temperature, and tidal amplitude data can help illustrate how the complex patterns of the interacting variables selected in the GAMM might occur. Three 10% sites were chosen for this example, Kildare, Bideford, and Enmore (Fig. 4). Kildare has the most extended periods of anoxia of any estuary (Fig. 4A). Supersaturation was observed at this site in the early months, while hypoxia extended well into the fall months contributing to the high frequency of zero values. In both cases, high oxygen and low oxygen occurred not only at warmer temperatures, but also at temperature below 10 °C. Thus, the trends in oxygen were opposite at different times of the year, hence the temperature-Julian day interaction. It can be observed that with extended periods of anoxia, tide had little or no influence on this for the duration of the year as oxygen was zero for much of the time. Bideford is in contrast to this in that it did not become supersaturated with oxygen but did experience cyclical periods of hypoxia, contributing to the bimodal pattern in oxygen frequency (Fig. 4B). Those cyclical periods of hypoxia did relate to the spring-neap tidal cycles for portions of the summer, but tide had no influence on oxygen during the shoulder seasons, nor did temperature consistently relate to oxygen, hence a complex interaction between tide, temperature, and Julian day. Enmore was the most normoxic of the examples used here (Fig. 4C) and had comparatively small changes in oxygen over a relatively narrow range linearly related to temperature and tide (Fig. 4C, Table 3).

## Discussion

Successful monitoring of eutrophication in estuaries requires detailed knowledge of the spatial and temporal variation of monitoring endpoints, their interrelationships, and the factors that affect variability. In this study, high-frequency dissolved oxygen data were acquired in the more heavily nutrient-impacted upper estuary and farther downstream where normoxic conditions were more common. Correlations between upper and mid-estuary dissolved oxygen concentrations were not as strong as anticipated as they became increasingly decoupled throughout the season. Complex relationships were found between oxygen, temperature, chlorophyll, daily tidal amplitude range, and day of the year that were specific to each estuary and each station within an estuary highlighting the importance of a variety of factors on dissolved oxygen concentration.

Nutrient effects that manifest through oxygen levels were most pronounced in the upper estuary and were initially well-correlated to the mid-estuary, though a temporal disconnect between these locations developed as the season progressed. These findings are consistent with southern European findings from Iriarte et al. (2010, 2015) who also suggest that the outer estuary may be more affected by local factors than nutrient loading from freshwater. As such, our initial hypothesis that monitoring at a single station in the upper estuary will be predictive of dissolved oxygen dynamics in the rest of the estuary is not fully supported. Although oxygen input and export of nutrients via organic matter in the upper estuary potentially influence oxygen concentrations further down the estuary, the relationship is not straightforward due to the influence of other factors such as water residence time, light limitation for benthic production, and local nutrient availability (Lavaud et al., 2020). Longer water residence time, a function of both lower tidal flushing and less freshwater forcing, reduces the importation of oxygen into the estuary and increases the likelihood of hypoxia or anoxia (Coffin et al., 2018a, b; DFO, 2021). Within-estuary differences due to residence time variability examined here are far less influential than between estuaries where differences in tidal amplitude are more pronounced (Coffin et al., 2018a, b). The decoupling of oxygen within estuaries suggests that local conditions, such as internal nutrient release, have a stronger influence on oxygen. The manifestation of phytoplankton blooms after hypoxic or anoxic events also indicates the importance of local nutrient release from organic matter. The role of internal nutrient cycling was not investigated here, but in other shallow eutrophic systems, it can be either a major or minor nutrient source (Bukaveckas & Isenberg, 2013; Valdemarsen et al., 2015). The consumption of labile organic carbon in sediments is rapid. Carbon dioxide efflux experiments show an exponential decay in organic carbon utilization leading to depletion within 50 days, supporting the reasoning for oxygen decoupling herein (Valdemarsen et al., 2014). Resolving these issues involves incorporating biogeochemical processes associated with sediment-surface water interactions into estuarine monitoring.

The decoupling of upper- and mid-estuary dissolved oxygen observed after August was likely due to the exhaustion of labile organic matter, or more specifically, nutrients internally derived from organic matter at the mid-estuary location. In estuaries that experienced low-oxygen, spatial decoupling typically occurred earlier in the season. Low oxygen was more severe in the upper estuary as the rate of primary production is highest in this area (Coffin et al., 2018a, b; Lavaud et al., 2020), specifically for estuaries in this study but in general for estuaries not characterized by high turbidity in the upper estuary or estuaries with a low proportion of nutrient loading from point-sources discharging into the estuary itself, e.g., municipal sewage or industry. It has been previously observed that virtually all of the nutrient load from freshwater, i.e., land-based sources, is consumed within a short distance from the transition zone (Valiela et al., 2021). Higher chlorophyll levels in the upper area of the estuary, seen herein, support this contention, meaning that only a fraction of the total inorganic nitrogen from the main riverine input load reaches the mid-estuary. As nutrient availability declines with distance from the upper estuary primary producers, there have a reduced opportunity for growth compared to the upper estuary and are therefore more dependent on the release of nutrients from local sources, e.g., sediment decomposition processes. Nutrient spiraling, downstream transport of nutrients from the upper estuary, occurs when there is a net flow of water; however, denitrification in the outer estuary counteracts this effect (Howarth et al., 2011). Consequently, a biomass increase in the outer estuary to a level where symptoms of eutrophication are evident, i.e., depressed dissolved oxygen from increased biological oxygen demand from decomposition and respiration from decomposers, takes longer than in the upper estuary. Furthermore, differences in denitrification rates may be quite different between the upper and outer estuary as sediments become increasingly sulfidic under anoxia which promotes sulfur oxidizing bacteria that in turn reduce denitrification (Howarth et al., 2011), exacerbating nutrient effects in the upper estuary.

The susceptibility to nutrient impacts is also diminished moving down the estuary since imported oxygen-rich ocean water is first mixed with water at outer-estuary locations and then the upper estuary (Lake & Brush, 2015). However, the physical characteristics of the estuary can also influence the importation of oxygen depending on whether there is water column stratification (Howarth et al., 2011). Taken together these mechanisms could both also contribute to the truncated period of low dissolved oxygen observed in the outer estuary. At the rare locations where the outer half of the estuary experienced anoxia in this study, anoxic periods did not always coincide with the upper estuary, indicating some degree of spatial independence. Conversely, Enmore, the most normoxic study site, had relatively linear relationships between available physicochemical variables that were best represented by our model. Thus, it seems that simple models can work well in systems with limited nutrient impact but that more mechanistic and spatially resolved modeling is required as system complexity and nutrient impacts increase.

Indeed, some form of ecosystem-based model may be needed to resolve the relationship between oxygen and spatial locations within an estuary. This could be accomplished through the increased collection of dissolved oxygen data spatially throughout an estuary, refined estimates for nutrient availability (both exogenous and endogenous and accounting for uptake by plants and low-trophic animals), bioturbation, and coupling with a spatially explicit hydrodynamic model at minimum (Clements & Comeau, 2019; Lavaud et al., 2020; Politi et al., 2021).

Tidal amplitude changes over the lunar cycle had some estuary-specific effects on dissolved oxygen but were not a dominant modifying factor of dissolved oxygen across all estuaries. This contrasts with research done across estuaries, at an annual time step, using cumulative oxygen metrics where tidal amplitude explained the majority of the variability between estuaries (Coffin et al., 2018a, b). The lack an overall relationship between tidal amplitude and dissolved oxygen herein can be explained by the differing magnitude of tidal effect within the upper estuary compared to between estuaries and timescale in the present study. Hypoxia or anoxia is an oxygen deficit, and tides function to import oxygen-rich ocean water into the estuary. However, in heavily nutrient-impacted situations, the increased flushing from neap to spring tides is not sufficient to compensate for the oxygen demand in upper to central parts of the estuary, and the net oxygen remains at zero, apparently uninfluenced by tidal changes in the short term as seen clearly in the Kildare estuary. This has been confirmed elsewhere through artificial oxygenation as an alternative to tidal oxygen transport, resulting in increases in dissolved oxygen in the water (Larsen et al., 2019). At some estuaries in the present study, the oxygen consumption was presumably less severe, and in the case of Bideford where cyclical hypoxia was observed seems to have been overcome by increased flushing. The third scenario is that oxygen is never in deficit, and therefore changes in tidal amplitude also have little effect (e.g., Enmore estuary). For this reason, the effects of tides were only observed at the low to moderately impacted stations. On a more regional scale as examined by Coffin et al. (2018a, b), differences between estuaries in tidal amplitude (a proxy for flushing) varied by tenfold, much greater than any within estuary difference herein. Metrics evaluated by Coffin et al. (2018a, b) were cumulative for the year (May to November), and at this timescale, cumulative hours of hypoxia or anoxia are likely to change due to the duration of the hypoxic/anoxic events. At shorter time scales as examined herein, tidal amplitude change appears to have no influence on impacted systems because when oxygen consumption is always higher than importation or photosynthesis, oxygen concentration will always be near zero and thus has limited variability. Thus, severity can only be measured in terms of the duration of anoxia over a longer period as per Coffin et al. (2018a, b).

Temperature did not have a consistent influence on oxygen levels in our model, despite obvious effects of temperature on dissolved oxygen through biological activity (increasing oxygen demand from respiration or oxygen production via photosynthesis) and through physical processes (dissolved oxygen solubility). Herein, the chemical effects of dissolved oxygen solubility were removed by correcting dissolved oxygen to a constant temperature. Rates of marine sediment nutrient cycling have been observed to increase significantly for phosphorus between 16 and 26 °C, though the largest increases for nitrogen occurred between 22 and 26 °C (Sans-Lazaro et al., 2015). A study using denitrification as a mineralization endpoint showed that activity increases tenfold between 5 and 18 °C (Nowicki, 1994). In the present study, the time variable in the GAMM analysis was significant more often than temperature, suggesting that seasonal processes are occurring with regard to oxygen consumption that do not correlate well with the annual temperature cycle. This is understandable as macroalgal growth/blooms that influence supersaturation do not often occur at the highest temperature, but in the spring when light penetration is greater due to higher water clarity (Schein et al., 2013). Phytoplankton blooms as seen herein can occur later in the year after anoxic events as nutrients are released, but water temperatures are getting cooler (Schein et al., 2012). While hypoxia did not appear to initiate until the water reached 15 °C, anoxic events have been observed at temperatures as low as 10 °C in some estuaries (Roloson et al., 2021). The implications from these findings are two-fold: (1) increasing temperature resulting from climate change may not further exacerbate internal-nutrient-based hypoxia in the ice-free season in contrast to the finding of others (Lajaunie-Salla et al., 2018). The decoupling of locations within an estuary, potentially due to limitations in labile sediment organic matter, emphasizes this point. Thus, temperature alone would be the most significant factor affecting temperature-sensitive species (Talmage & Gobler, 2011). (2) Temperature and precipitation changes due to climate change will also have distal impacts on eutrophication that could be more significant than internal loading changes. For example, summer stream flows are predicted to increase in pessimistic climate scenario (Charron et al., 2021), which, given cold groundwater-dominated nutrient sinks on PEI, could lead to higher external nutrient loading and during the summer months relatively colder water temperatures (Danielescu & MacQuarrie, 2011; Knysh et al., 2016).

One of the primary objectives of this study was to determine if monitoring dissolved oxygen in the upper estuary alone would provide sufficient information regarding dissolved oxygen at other spatial scales. While there was connectivity among locations, monitoring in multiple places along the gradient from upper to outer estuary will be critical if the objective is to ascertain the spatial extent of hypoxia, which has direct implications for shellfish aquaculture. Accordingly, a recommendation from this work is to maintain monitoring at two locations, 10% and 50%, at a minimum and at additional stations in between if the objective is to map the extent of hypoxia. While chlorophyll appeared to be indirectly linked to oxygen, inconsistent timing of blooms between estuaries, the lack of the ability to capture macroalgal production, and the poor performance of fluorescence-based continuous monitoring loggers in highly turbid estuary environments make oxygen a superior monitoring endpoint (James, 2013). A clearer understanding of dissolved oxygen spatial dynamics and its relationship with nutrient loading is critical for management decisions as hypoxia/anoxia can have dramatic influence over animal behavior (Roloson et al., 2021) and/or survivorship (Coffin et al., 2018b; Coffin et al., 2021). Currently only external nutrient loading is considered in the region (Bugden et al., 2014; Coffin et al., 2018a, b). The results of strong local influences observed herein suggest that internal loading of nutrients from sediment will be as, or more, important in predicting adverse oxygen events throughout the estuary. To meet this objective will likely require direct observation and quantification of those internal loadings as well as the development of ecosystem-based models that are able to integrate primary and secondary production with nutrient cycling spatially.

## Supplementary information

Below is the link to the electronic supplementary material.

Supplementary file1 (DOCX 253 KB)

## Data Availability

All data included in this publication can be made available upon request.
