# Supplementary material for: Influence of nutrient enrichment on temporal and spatial dynamics of dissolved oxygen within northern temperate estuaries
Source: Environ Monit Assess. 2021 Nov 15;193(12):804. doi: 10.1007/s10661-021-09589-8 (PMC8593059; doi:10.1007/s10661-021-09589-8)
Supplement: Supplementary file 1 — Supplementary file1 (DOCX 253 KB) [file 10661_2021_9589_MOESM1_ESM.docx]

Table S1. Parametric parameter estimates for individual GAMM models predicting oxygen smooths, for all intercepts p<0.001.

| Location | 10% | | 50% | |
| --- | --- | --- | --- | --- |
| **Estuary** | **Function** | **Intercept ± Standard Error** | **Function** | **Intercept ± Standard Error** |
| Wheatley | Oxygen ~ s(Julian Day) + s(Temperature) | 9.53±0.426 | Oxygen ~ s(Julian Day) | 9.14 ±0.181 |
| Mill | Oxygen ~ s(Julian Day) + s(Temperature) | 9.53±0.332 | Oxygen ~ s(Julian Day)+ s(Tidal Amplitude) | 8.96 ±0.485 |
| Kildare | Oxygen ~ te(Julian, Temperature) | 7.61 ± 3.581. | Oxygen ~ s(Julian Day) | 8.03 ±0.323 |
| Enmore | Oxygen ~ s(Temperature) + s(Tidal Amplitude) | 9.20 ±0.2.32 | Oxygen ~ s(Temperature) | 10.15 ±0.291 |
| Bideford | Oxygen ~ te(Julian, Temperature, and Tidal Amplitude) | 8.65 ±1.11 | Oxygen ~ s(Julian Day) | 10.00 ±0.196 |


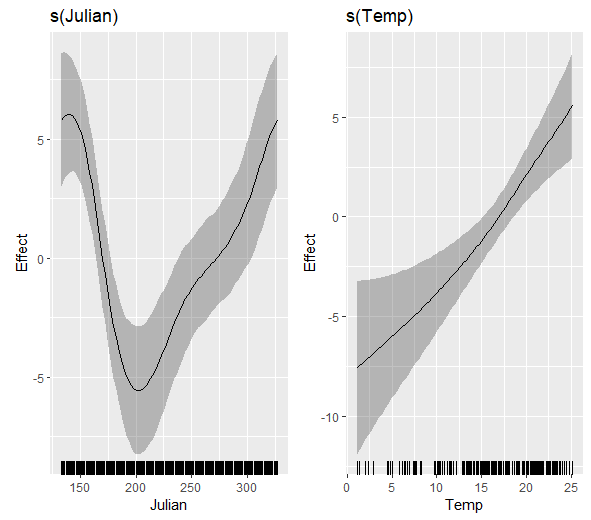


Figure S1. Fitted smooths ± 95 Confidence interval (CI) of oxygen concentration at the 10% location of the Wheatley estuary. Temp =Temperature °C, Julian =Julian Day.


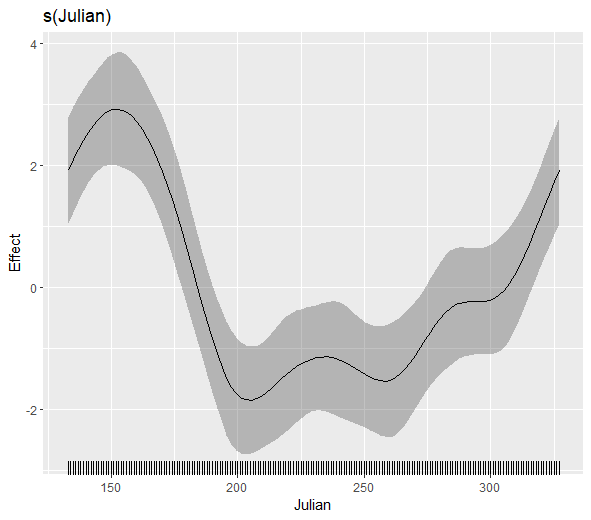


Figure S2. Fitted smooth ± 95 %CI of oxygen concentration at the 50% location of the Wheatley estuary.


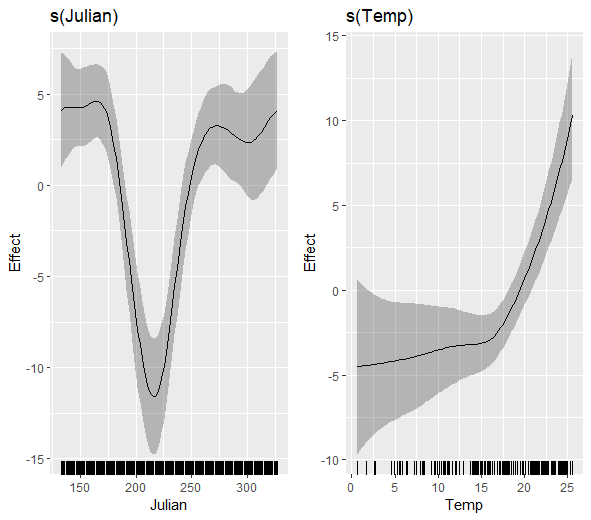


Figure S3. Fitted smooth ± 95 %CI of oxygen concentration at the 10% location of the Mill estuary.


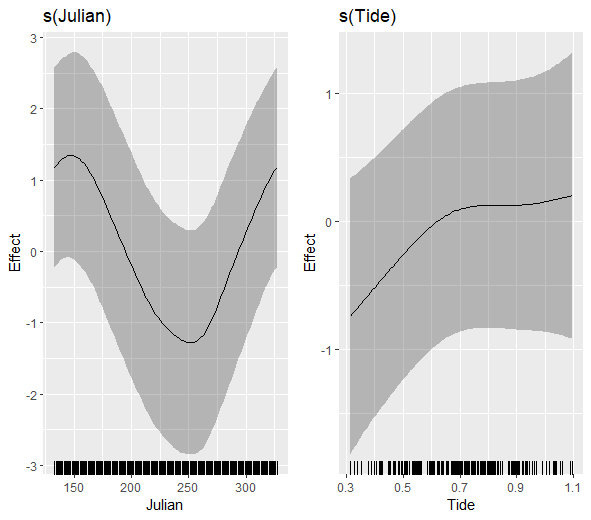


Figure S4. Fitted smooth ± 95 %CI of oxygen concentration at the 50% location of the Mill estuary. Tide = Tidal Amplitude (m).


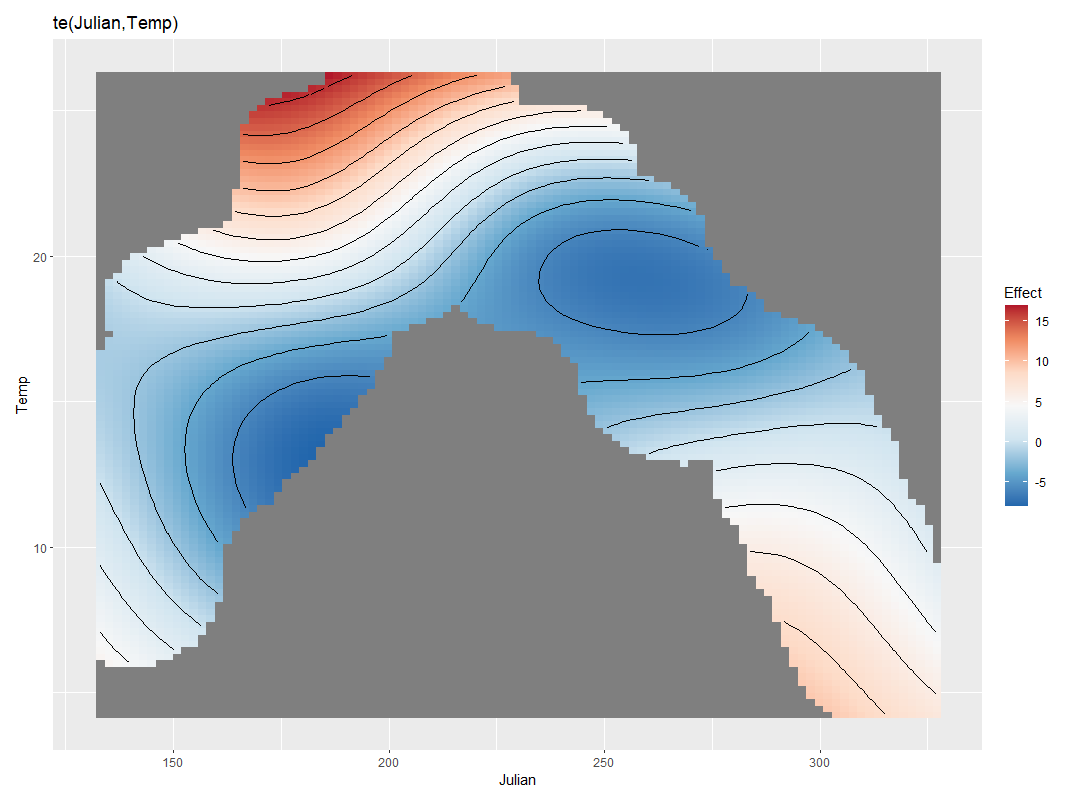


Figure S5. Fitted tensor interaction smooth of oxygen concentration at the 10% location of the Kildare estuary.


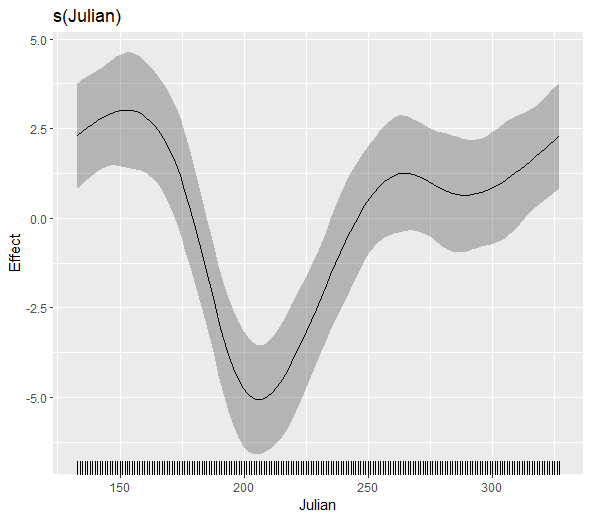


Figure S6. Fitted smooth ± 95 %CI of oxygen concentration at the 50% location of the Kildare estuary.


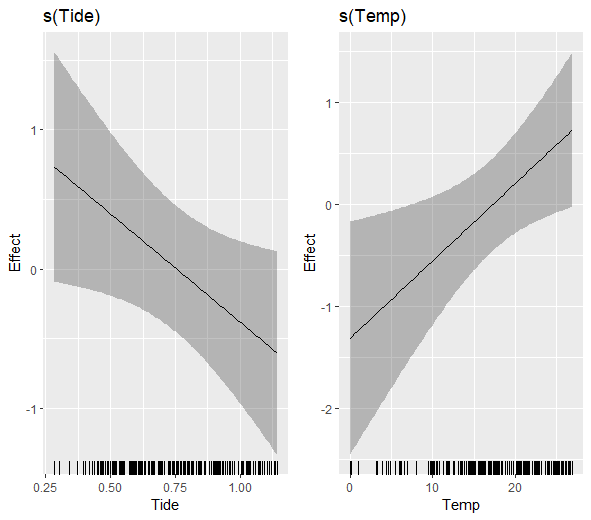


Figure S7. Fitted smooth ± 95 %CI of oxygen concentration at the 10% location of the Enmore estuary.


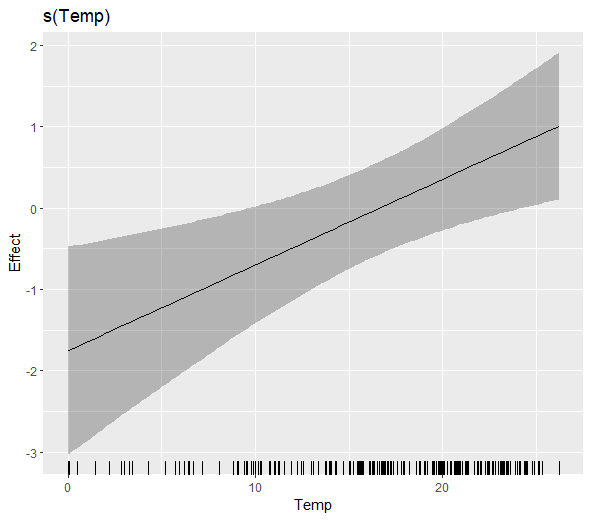


Figure S8. Fitted smooth ± 95 %CI of oxygen concentration at the 50% location of the Enmore estuary.


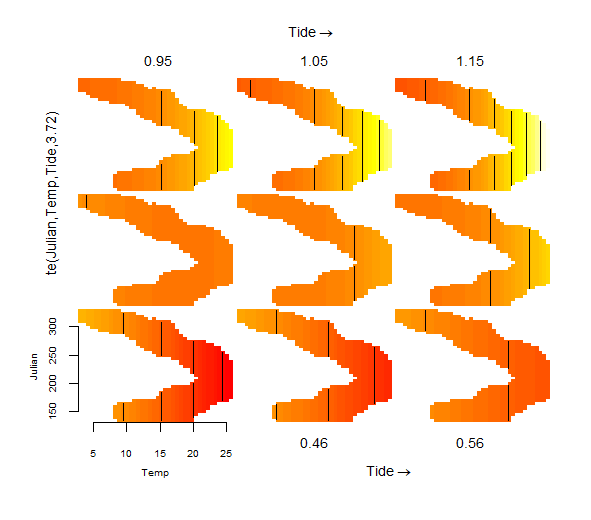


Figure S9. Fitted tensor interaction smooth of oxygen concentration at the 10% location of the Bideford estuary.


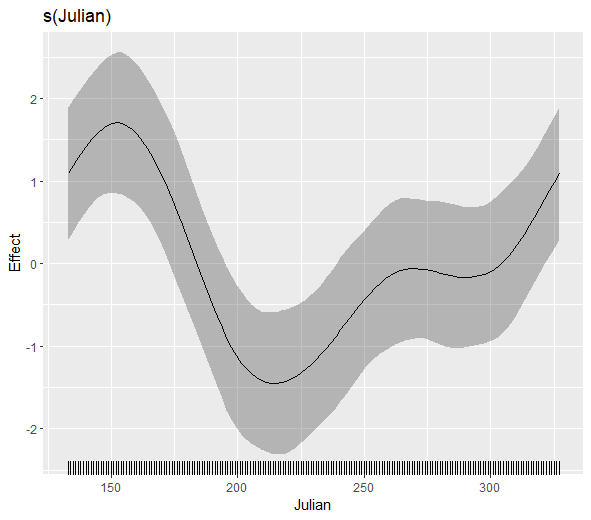


Figure S10. Fitted smooth ± 95 %CI of oxygen concentration at the 50% location of the Bideford estuary.
